# The Effective Phonon Frequency and Mass Involved in Electron–Phonon Coupling at Metal Surfaces from Helium Atom Scattering

**DOI:** 10.1021/acs.jpclett.6c01733

**Published:** 2026-09-01

**Authors:** J. R. Manson, G. Benedek, S. Miret-Artés

**Affiliations:** † Department of Physics and Astronomy, 2545 Clemson University, Clemson, South Carolina 29634, United States; ‡ Donostia International Physics Center (DIPC), Paseo Manuel de Lardizábal 4, 20018 Donostia−San Sebastián, Spain; § Department of Materials Science, University of Milano-Bicocca, Via R. Cozzi 55, 20125 Milano, Italy; ∥ Instituto de Física Fundamental, Consejo Superior de Investigaciones Científicas, Serrano 123, 28006 Madrid, Spain

## Abstract

In this work, we propose a new formulation of the classic Debye–Waller theory for the scattering of He atoms from metal surfaces. The probe atoms are repelled by the surface free-electron density just above the first surface atomic layer and the modulation of the surface charge density produced by lattice vibrations occurs via the electron–phonon coupling. In the Debye–Waller exponent, the surface-projected phonon spectrum is reduced to that of an effective single phonon branch responsible for the total electron–phonon coupling. This is characterized by an effective surface phonon frequency and an effective surface atom mass, which are extracted from the high and low surface temperature limits. Whether an acoustic or optical phonon branch is considered, a Debye or Einstein model for the surface can be assumed, respectively, leading to simple relationships between the effective parameters. The Einstein model is shown to work well for the copper surfaces (110), (113), (001) and (111), according to the HAS data reported by Lapujoulade et al., while for the clean and oxidized Nb(001) surfaces, the analysis of the HAS data recently reported by Arias, Sibener et al., suggests a dominant role of the surface acoustic phonons in the surface electron–phonon interaction.

## Graphical abstract

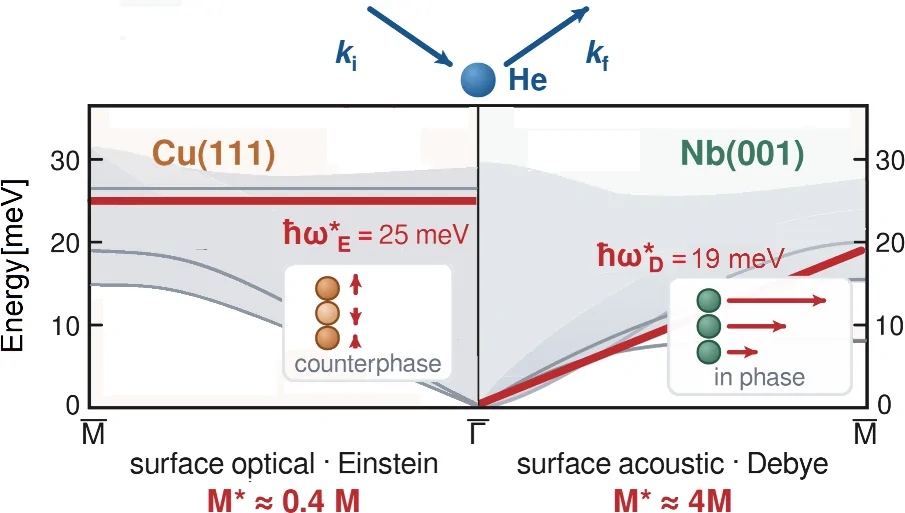

In the scattering of neutral atoms from a crystal surface all quantum mechanical features, such as the diffraction peaks from a periodic surface, the diffuse elastic features due to scattering from imperfections and defects, or inelastic scattering from single-phonon peaks, are thermally attenuated by a Debye–Waller (DW) factor.^1^ The attenuation of the scattering intensity I­(T) has the form

1 $$I\left(T\right)=I_{0}e^{-2W\left(\Delta k,T\right)}$$

where T is the surface temperature, 2*W*(Δ**k**,*T*) the DW exponent, with Δ**k** = **k**_*f*_ – **k**_*i*_ the difference between the wavevectors of the scattered and incoming atomic projectile. The DW exponent, in the classic form valid for a direct interaction of He atoms with the surface atoms, reads as

2 $$2W\left(\Delta k,T\right)={\left\langle {\left(\Delta k\cdot u_{0l}\right)}^{2}\right\rangle }_{T}$$

where the angular brackets ⟨···⟩_*T*_ mean the thermal average over the ensemble of phonon states, and u_0*l*_ is the thermal vibrational displacement of the surface atom (index 0) in the *l*-th unit cell of the surface. With the assumption that each single atomic layer parallel to the surface is a Bravais lattice with one atom in the 2D unit cell, no other atom index is needed, and the thermal average is independent of index *l*. According to eq 2, I_0_ gives the scattering intensity for a rigid surface (u_0*l*_ = 0). It is therefore independent of temperature and larger than the experimental I(0) because of the atomic zero-point motion. The temperature dependence of the diffraction peak intensity comes entirely from that of the Debye–Waller exponent.

In the classic theory of atom scattering from a crystal surface the DW exponent can be evaluated in much the same way as for neutron or X-ray scattering from the bulk,^2,3^ except that there the mean-square displacements are those of the surface atoms. As shown in refs 4 and 5 , it is found that

2W(kf,ki;T)=∑αα′ΔkαΔkα′∑Qνe0,α+(Q,ν)e0,α′(Q,ν)×ℏ2NMω(Q,ν)coth⁡ℏω(Q,ν)2kBT 3

where α,α′ label the Cartesian components of the scattering vector Δ**k** and the phonon polarization vectors *e*_0.α_(**Q**,ν) of the surface atoms for the surface wavevector **Q** and phonon branch ν, N is the number of phonon modes, and M the surface atom mass. The dependence of the DW exponent on the surface temperature T is given by the hyperbolic-cotangent (coth) Bose–Einstein factor, where k_B_ is the Boltzmann constant. The sum over the phonon eigenmodes can be rewritten as

2W(kf,ki,T)=ℏ22M∑αα′ΔkαΔkα′×∫0ωmaxdωρ0,αα′(ω)ℏωcoth⁡ℏω2kBT 4

where ρ_0,*αα*′_(ω) is the surface-projected phonon density matrix (ref 6 Chapter 7) and ω_max_ the maximum frequency of the phonon spectrum.

This formulation of the DW exponent, eq 4, where the He atom transfers a momentum *ℏΔ* ***k*** to surface atoms through direct two-body collisions, is generally appropriate to insulator or semiconductor surfaces, but not for He atoms scattering (HAS) from metal surfaces. In general, for a metal surface the probe He atoms are repelled by the surface free-electron density a few Å above the first surface atomic layer. Thus, the dynamic modulation of the surface charge density, and therefore of the He-surface scattering potential induced by atomic thermal vibrations near the surface, only occurs via the electron–phonon (el-ph) coupling.^7,8^ This finding suggested that information on the el-ph coupling constant (mass-enhancement factor) at the metal surfaces can be directly obtained from the temperature dependence of the elastic HAS DW exponent.^9–15^

It is therefore interesting to adapt eq 4 to the case of metal surfaces, where the el-ph coupling confers a many-body character to the He-surface atom interaction, by involving some specific phonon branch, which plays a dominant role in that interaction. Such extension of eq 4 is here achieved by reducing the surface-projected phonon spectrum to that of a single phonon branch with the largest el-ph interaction, and fully characterized by an effective frequency ω* and an effective atom mass M*. In this way, the effective frequency and mass can be directly extracted from the high and zero temperature limits of the experimental DW exponent. In the classic theory of the DW factor for a monatomic crystal, only an acoustic phonon branch with a linear Debye-like dispersion was considered, so that ω* is just the Debye frequency and M* = M.

For a semi-infinite monatomic lattice with an unreconstructed surface, the unit cell defined by the two surface basis vectors and the third one normal to it, always includes more atoms than just the one on the first layer (with the exception of the simple-cubic (001) surface). For example, a monatomic face-centered cubic crystal surface with the 3D unit cell spans two surface atomic planes (2 atoms per unit cell) for the (001) and (110) surfaces, and three surface atomic planes (3 atoms per unit cell) for the (111) surface. Thus, surface-localized optical phonon modes are expected, in which the two (three) atoms of the unit cell move antiphase with a fairly flat, Einstein-like dispersion, so as to keep, at zero wavevector, the center-of-mass fixed. In this case the effective mass is actually the reduced mass of the two (three) atoms of the unit cell, M* < M, while the frequency of the flat Einstein branch, providing alone the dominant el-ph coupling, shall be termed effective Einstein frequency (ω* ≡ ω_E_^*^). It is noted that the antiphase motion of the atom cores in the unit cell causes a modulation of the free electron density, which is larger than that produced by the more-in-phase motion of the cores in the acoustic modes. This would yield a larger el-ph coupling for the flat surface optical phonon branches, thus justifying the use of the Einstein approximation. On the other hand, the displacement field of the surface nearly transverse acoustic modes, such as the Rayleigh wave (RW), decays exponentially below the surface layer, with a decay length proportional to the surface phonon wavelength, and a dephasing between the first and second layer which increases toward the surface Brillouin zone boundary. Thus, also RWs can produce significant surface charge-density oscillations, suggesting a fit based on the Debye model. Due to the nonoscillatory exponential decay of the displacement field encompassing a few layers, an effective mass M* > M is expected.

In the following, available HAS data on the temperature dependence of the DW exponent for different surfaces of copper^16,17^ and for the clean and oxidized Nb(001) surfaces^18–21^ are analyzed within both the Einstein and the Debye model. While for the copper surfaces the surface optical phonons are seen to provide the major contribution to the electron–phonon interaction, for Nb(001) this contribution is seen to be mostly provided by the surface shear-vertical (SV) acoustic branch (actually the RW), in agreement with Méndez et al. ab initio density-functional calculations.^18–21^

The Einstein surface-projected phonon density matrix is simply written as

$$\rho_{0,\alpha \alpha \prime }^{E}\left(\omega \right)≃\delta_{\alpha \alpha \prime }\;\delta \left(\omega -\omega_{E}^{*}\right)$$ 5

whereas the phonon density of states for the Debye model, consisting of a single linear phonon dispersion curve starting from zero energy and momentum, and suitable therefore to describe acoustic phonon branches, reads

$$\rho_{0,\alpha \alpha^{\prime }}^{D}\left(\omega \right)≅3\delta_{\alpha \alpha^{\prime }}\omega^{2}/{\left(\omega_{D}^{*}\right)}^{3}\;\mathrm{for}\;\omega \leq \omega_{D}^{*}$$ 6

where ω_D_^*^ is the effective Debye frequency.

In the general case of specular elastic scattering (Δ**k** = (0,0,2*k*_*iz*_)), by replacing ρ_0,*αα*′_(ω) in eq 4 with its Einstein form, eq 5, and ω_max_ > ω_E_^*^, it is found that

7 $$2W_{E}\left(\Delta k,T\right)=\frac{{\left(2\hbar k_{iz}\right)}^{2}}{2M_{E}^{*}{\hbar \omega }_{E}^{*}}\;\coth\frac{\hbar \omega_{E}^{*}}{2k_{B}T}$$

with the limits of 2*W*_*E*_(Δ ***k***,*T*) for T→0 and *T* ≫ *T*_*E*_ ≡ *ℏ*ω_E_^*^/*k*_*B*_ given by

8 2WE(0)=2(ℏkiz)2ℏωE*ME*(8a)2WE(T≫TE)≅2kBT2(ℏkiz)2(ℏωE*)2ME*(8b)

With these expressions, the effective Einstein phonon frequency *ℏω*_E_^*^ and mass *M*_E_^*^ can be directly obtained from the limiting values of the DW exponent, measured as a function of the surface temperature:

9a $$\hbar \omega_{E}^{*}≅\frac{2W_{E}\left(0\right)2k_{B}T}{2W_{E}\left(T\gg T_{E}\right)}$$

9b $$M_{E}^{*}≅\frac{2{\left(\hbar k_{iz}\right)}^{2}}{2W_{E}\left(0\right)\hbar \omega_{E}^{*}}$$

Similar expressions are obtained for the effective frequency ω_D_^*^ and mass *M*_D_^*^ in the Debye approximation for the phonon spectrum, when ρ_0,*αα*’_(ω) in eq 4 is given the Debye form, eq 6, with ω_max_ = ω_D_^*^. This gives simple relationships between the effective parameters:

10 $$\omega_{D}^{*}=2\omega_{E}^{*},M_{D}^{*}=\frac{3}{4}M_{E}^{*}$$

As appears from eq 9b, the effective mass derived from the experimental DW exponent at eq 3 depends explicitly on the z component of the incident momentum at the contact point with the surface charge density. It is therefore necessary to correct for the kinetic energy increase when the He atom is approaching the classical turning point under the action of the surface attractive potential (Beeby correction^26^). This is done by replacing (*ℏ* *k*_*iz*_)^2^ with

11 $${\left(\hbar {\mathrm{k̅}}_{iz}\right)}^{2}={\left(\hbar k_{iz}\right)}^{2}+2m_{He}D$$

The specular elastic HAS intensity as a function of the surface temperature for the (110), (113), (001), and (111) surfaces of copper, as measured by Lapujoulade et al.,^16^ are reproduced in Figures 1a,c,e and 3a. The wavevector *k*_i_ of the incident He beam and its polar angle θ_i_ with respect to the axis z normal to the surface are given in the figures and Table 1. These experimental data, reported in ref 16 up to 800 K, are here reproduced in Figure 1 below 500 K, because the (110) and (113) surfaces exhibit a roughening phase transition above T_R_ ≈ 500 K, with a sudden increase of the 2W­(T) slope.^16,27^ The el-ph interaction in the surface roughened phase and its signatures in the T-dependence of the DW exponent are not considered here, though definitely deserving a future investigation.

**Table 1:** Normal Wavevector (Column 2) and Polar Angle (Column 3) of the Incident He Beam in HAS Experiments on Different Copper Surfaces^16,17^ and on Clean and Oxygen-Covered Nb(001) Surfaces,^18–21^ and the Surface Potential Depth D (Column 4) Used in the Derivation of the Effective Frequency (Column 5) and Effective Mass (Column 6, To Be Compared with the Cu Atom Mass of 63.55 a.u. and the Nb Atom Mass of 92.9 a.u.)^a^

Surface	*k*_iz_ [A^–1^]	θ_i_ [deg]	D [meV]	$\hbar \omega_{E}^{*}=\frac{1}{2}\hbar \omega_{D}^{*}$ [meV]	*M*_E_^*^ [a.u.]	Surface phonon theory
Cu(110)	6.35	20	6.35^a^	22.1	47.0	EAM^d^
Cu(113)	6.35	20	6.35^a^	19.1	58.4	MBEP^e^
Cu(001)	6.35	55.5	6.35^a^	23.8	32.1	MBEP^e^
		31.8		23.8	31.3	
Cu(111)	11.0	56.9	9.7^b^	25.0	26.1	DFPT^f^
Nb(001)	5.20	35	5.8^c^	*ℏ*ω_D_^*^ = 19	*M*_D_^*^ = 380	DFT,^g^ FC^h^
(3 × 1)-O/Nb(001)	4.82	35	4.7[Table-fn t1fn9]	*ℏ*ω_D_^*^ = 22	*M*_D_^*^ = 107	DFT^g^

a The last column lists the theoretical methods used in the calculation of the surface phonon dispersion curves to be compared with the phonon branches mostly contributing to the temperature dependence of the DW exponent.

b Reference 16.

c Reference 17.

d Reference 21.

e Embedded atom method.^23^

f Many-body empirical potential.^24^

g Density functional perturbation theory.^25^

h Density functional theory.^18,21^

I Force-constant model fit.^22^

j Reference 33.

**Figure 1:**
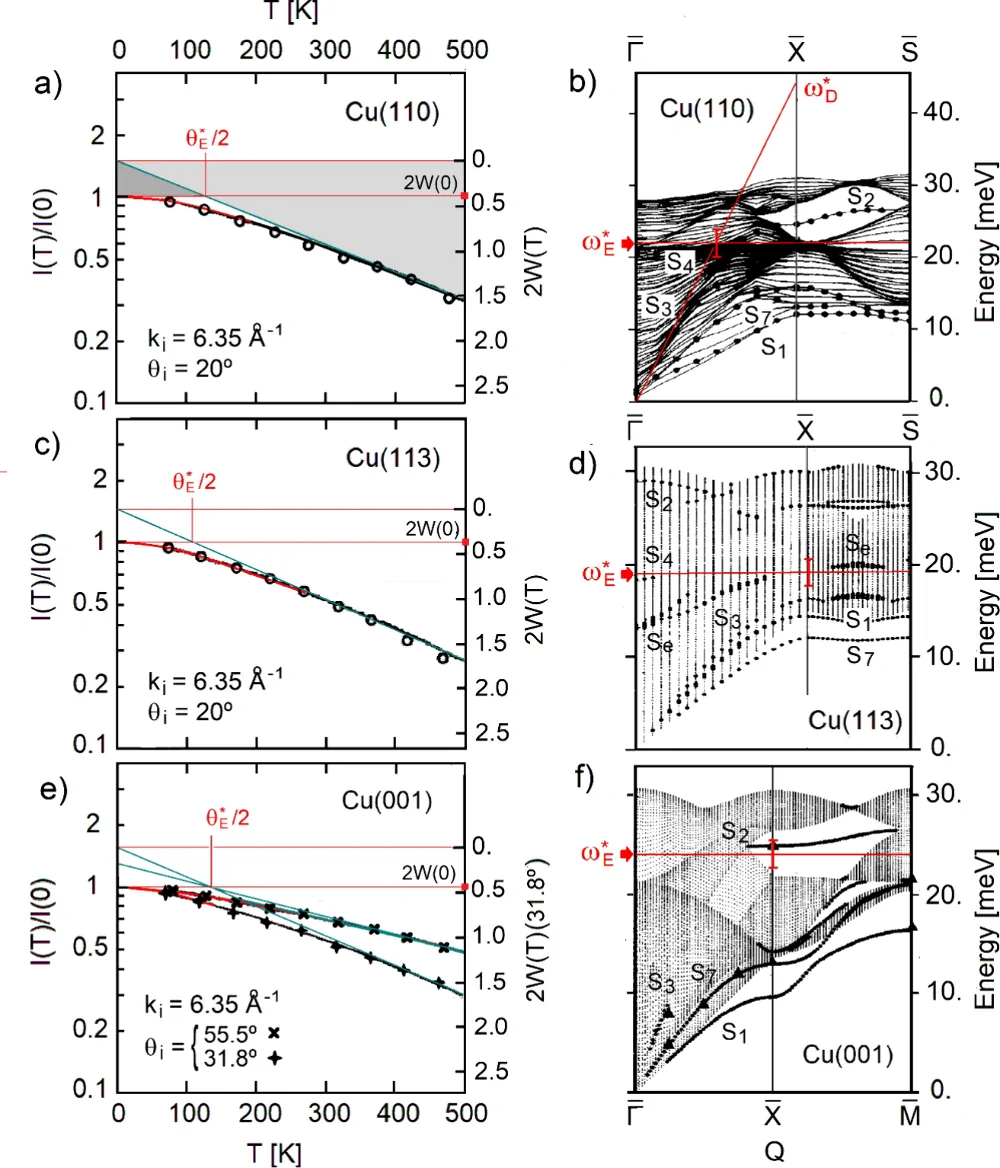
(a) The He atom scattering intensity of the elastic specular peak (relative to its zero-temperature value, in a log scale) from the Cu(110) surface as a function of the surface temperature T, measured by Lapujoulade et al.^16^ (HAS incident wavevector *k*_i_ and polar angle θ_i_ are indicated in the low-left corner), and extrapolated to *T* = 0 through a hyperbolic cotangent (coth) fit. The high-temperature asymptotic straight line (green line) defines the zero of the Debye–Waller exponent 2W­(T) (right-hand ordinate linear scale). The value of 2W(0) is marked by a red dot. The abscissa of the intersection between the 2W(0) horizontal straight line and the coth asymptote gives one-half of the effective Einstein frequency in temperature units. (b) The Einstein and Debye dispersion lines (red), with the respective frequencies, superimposed to the calculated surface-phonon dispersion curves of Cu­(110),^18^ showing the correspondence with the surface phonons branches most contributing to the electron–phonon interaction. (c) and (d) Same as (a) and (b) for Cu(113) with experimental data (**o**) in (c) from ref 16 and the surface phonon calculation from ref 24. (e) and (f) Same as (a) and (b) for Cu(001) with two different sets of experimental data (**×**,**+**) in (e) from ref 16 and the surface phonon calculation from ref 24.

The hyperbolic cotangent fit of the data points with eq 7, represented in Figures 1a,c,e on a log scale (left-hand ordinate) permits to extrapolate the T = 0 scattered intensity I(0) (red curve). On the log scale, the asymptotic straight line (green line) of I­(T) at high temperature defines I_0_ at T = 0 and gives 2W(0) = ln­[I_0_/I­(0)]. I_0_ is the specular intensity reflected by an ideal rigid lattice surface, i.e., at T = 0 in the absence of zero-point vibrations. The same hyperbolic cotangent fit provides then 2W­(T) on a linear scale (right-hand ordinate). The value of 2W(0) is indicated on this scale by a red square.

It is worth noting that the actual value of 2W(0) is not really necessary. If the coth function of eq 7 is expanded in a Taylor series in its argument, each term in the series depends differently on the effective mass and the effective frequency. For example, including only the first three terms in such an expansion matches the exact expression quite well to all low experiment temperatures shown in Figure 1. Thus, fitting the data to the expansion is an alternate way of determining both the effective mass and frequency.

The similarity of the two rectangular triangles (different gray areas in Figure 1a permits to derive graphically one-half of the effective Einstein frequency in K units, θ_E_^*^ = *ℏ*ω_E_^*^/*k*_*B*_, according to the proportionality expressed by eq 9a. With *ℏ*ω_E_^*^ derived in this way, *M*_E_^*^ is then obtained from eq 9b, and the corresponding values in the Debye model from eq 10. The resulting values are listed in Table 1. The location of the resulting Einstein horizontal branch at *ℏω*_E_^*^ on the surface phonon dispersion curves, calculated with different semiempirical and ab initio methods (Table 1, last column),^18,21–25^ is shown in Figures 1b,d,f for Cu(110), Cu(113), Cu(001), respectively. For Cu(110), Figure 1b, the resulting Debye linear dispersion is also displayed. The red error bar on the Einstein and Debye dispersion lines originates from the estimated uncertainty in the coth fits of experiments.

It clearly appears that for all three surfaces the dominant el-ph interaction can be associated with surface optical phonon branches as follows:

For Cu(110), the Einstein-like surface phonon branch S_4_, extends from the center to the boundary of the surface Brillouin zone (BZ); interestingly, the Debye-like acoustic dispersion curve is superimposed in the lower part to the anomalous longitudinal resonance S_3_.

For Cu(113), the Einstein branch apparently agrees with the BZ center mode S_4_, while at the BZ boundary it is close to the edge branch S_e_ (Figure 1d), the quasi-one-dimensional phonon branch localized along the atom rows at the step edges of the (113) surface (Figure 2a,b).^17^ It is also possible that in the BZ central region the position of *ℏω*_E_^*^ reflects the combined el-ph contributions of the S_e_ and S_2_ surface phonon branches.

For Cu(001) the effective frequency *ℏω*_E_^*^ can clearly be associated with the zone-boundary gap surface mode S_2_, also known to have in fcc(001) metal surfaces a large el-ph interaction, associated with the ion longitudinal displacements in both the first and second surface atomic layers.^24,28,29^ It is interesting to note that the HAS data, reproduced in Figure 1e for two different incident polar angles of 55.5° and 31.8°, yield consistently (as it should be) the same *ℏω*_E_^*^ and, as discussed below, about the same effective mass.

**Figure 2:**
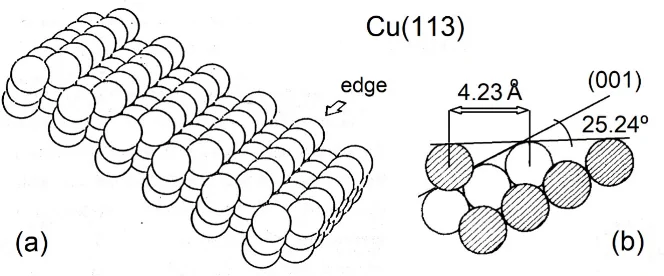
Cu­(113) vicinal surface in (a) a perspective view, showing the edge atom rows, and (b) a section normal to the edge direction.

The low temperature HAS data reported by Lapujoulade et al.^16^ for Cu(111) (Figure 3a) are not close enough to 0 K to allow for a sufficiently reliable cotangent fit with eq 7. In this case, ab initio calculations based on density functional perturbation theory (DFPT) (Figure 3b)^25^ indicate the Einstein-like S_2_ surface optical resonance, with the largest vertical amplitudes in the second surface layer, as providing a large el-ph coupling all over the BZ, together with the S_3_ anomalous longitudinal resonance, but only in the BZ boundary region. By associating *ℏω*_E_^*^ to the S_2_ branch, the data are used to extract the effective mass.

**Figure 3:**
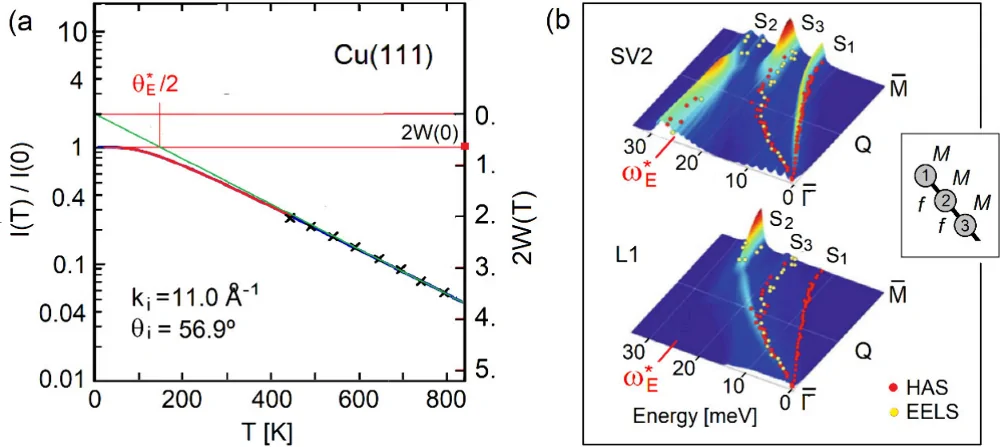
(a) Same as Figure 1a for Cu(111) with experimental data (×) from ref 17. (b) DFPT calculated surface-phonon density of states with shear vertical polarization, projected on the second surface layer (SV2) and longitudinal polarization projected on the first surface layer (L1) along the Γ̅Μ̅ symmetry direction of the wave vector Q.^20^ The HAS and EELS experimental data show an excellent agreement with DFPT calculation; the Einstein phonon branch derived from 2W­(T) corresponds very well to the S_2_ branch and fairly well also to the S_3_ resonance at the BZ boundary. The inset shows the trimer in the surface unit cell, where M is the copper atom mass and f the nearest neighbor force constant.

The effective mass obtained from eq 9b is listed for the four copper surfaces in Table 1, column 6 and has to be compared to the copper atomic mass M_Cu_ = 63.5 a.u. Qualitatively speaking, in a monatomic crystal with more atoms in the unit cell, their effective mass can be larger or smaller than the atom mass, depending on whether the atomic displacements of a given vibrational mode within the unit cell move mostly in phase or in counterphase. For example, in a diatomic lattice the effective mass of acoustic phonons tends to the sum of the two atom masses in the longwave limit, while that of optical modes tends in the longwave limit to the reduced mass of the unit cell.

Of the values of M* in Table 1, only that of Cu(113) is close to M_Cu_. This can be explained by the nature of the edge vibrational mode, propagating along a quasi-1D monatomic chain, for which the effective mass is just the atomic mass. On the other hand, the effective mass of the Cu(001) optical mode S_2_ is about one-half of M_Cu_ from both HAS measurements with the two different incidence angles. Since the monatomic fcc(001) surface is a pile of atomic bilayers made of diatomic surface unit cells, the two atoms of each unit cell (on two adjacent planes) oscillate counterphase in the S_2_ modes. Also the monatomic fcc(110) surface is made of atomic bilayers with diatomic surface unit cells; the fact that M*/M_Cu_ ≅ 3/4 suggests a less pronounced role in the el-ph interaction of the optical surface mode S_4_ and an enhanced participation of the acoustic longitudinal resonance S_3_ (Figure 1b).

Unlike the previous two surfaces, the monatomic fcc(111) surface is a pile of trilayers made of unit cells with three atoms, one per plane. In this case, the counterphase motion of the second surface plane against the motion of the first and third layers can give an M* smaller than M_Cu_/2. This can be argued from the dynamics of the three atoms of mass M in the surface unit cell (inset of Figure 2b) connected by the nearest neighbor force constant f. When the displacement of the atom 3 in the third layer is neglected in view of the strong surface localization of the S_2_ mode,^25^ the higher vibrational eigenvalue and the corresponding eigenvector ratio are found to be

12 $$\omega_{+}^{2}=f/M^{*},M^{*}=2M/\left(3+\sqrt{5}\right)≅0.382M,u_{1}/u_{2}=\left(1-\sqrt{5}/2\right)=-0.618$$

The good agreement of this effective mass with the experimental value given in Table 1, M* = 0.41M, speaks in favor of the model and approximations made in the present analysis.

It is now interesting to apply the above analysis to the available data for the Nb(001) surface.^18,21^ Niobium, with its superconducting critical temperature *T*_c_ = 9.25 K (the largest elemental *T*_c_ at ambient pressure^30^), has at the (001) surface a mass-enhancement factor (measured with HAS) λ_HAS_ = 0.50 ± 0.08.^20^ This is consistently much larger than the average λ_HAS_ = 0.11 ± 0.02 for the four Cu surfaces,^31^ which have no measurable *T*_c_. The HAS DW exponent 2W­(T) for the clean Nb(001) surface (Figure 4a), calculated ab initio by Méndez et al.^21^ and shown to provide an excellent fit to the high-temperature HAS data, yields, from eqs (9,10), *ℏ*ω_D_^*^ = 19 meV and*M*_D_^*^= 380 a.u. for the Debye model and *ℏ*ω_E_^*^ = 9.5 meV and *M*_E_^*^= 506 a.u. The effective masses are about 4 and 5.5 times larger than the Nb atomic mass of 92.9 a.u. for the Debye and Einstein models, respectively. The comparison of these values with the DFT surface phonon dispersion curves calculated by Méndez et al.^21^ (Figure 4b), shows a clear correspondence of *ℏ*ω_D_^*^ to the zone-boundary linear extrapolation of the acoustic SV (RW) branch, whereas *ℏ*ω_E_^*^ corresponds to the large flat portion of the surface longitudinal acoustic (SLA) branch. This is consistent with the fact that in Nb(001) no optical surface-localized mode occurs with an appreciable top-layer amplitude (Figure 4b,c), unlike at Cu surfaces, where the modest surface el-ph interaction mostly comes from the optical surface modes (Figures 1 and 2). Méndez et al.^21^ found that surface SV modes alone contribute for the 52.4% to the DW exponent, while SLA modes only contribute for the 7.4%.

**Figure 4:**
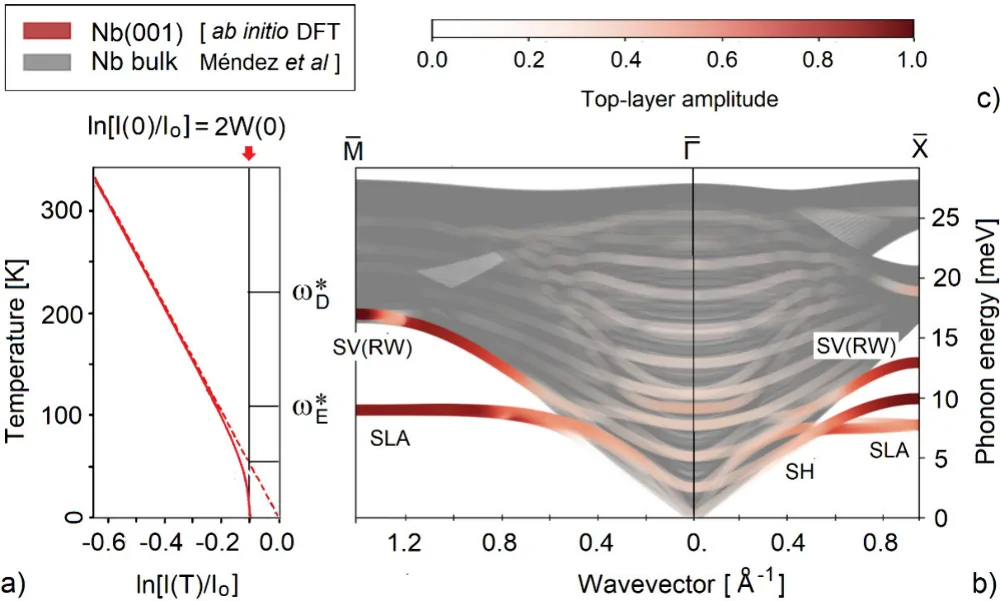
(a) Logarithm of the normalized HAS specular intensity (abscissa) from the Nb(001) clean surface as a function of temperature (ordinate) from Méndez et al.^21^ ab initio calculations for an incident energy of 21 meV (full line), providing an excellent fit of high-temperature DW experimental data.^21^ The linear extrapolation (broken line) defines the normalization intensity I_0_ and hence 2W(0) (red arrow). The corresponding effective Debye and Einstein frequencies are indicated in temperature units. (b) The latter effective frequencies are seen to correspond quite well to ab initio DFT calculations,^18,21^ as well as to the original force-constant model fits of, respectively, the Rayleigh-wave (RW) (shear-vertical (SV) polarized the zone boundaries) and the flat surface longitudinal acoustic (SLA) branch. The SLA branch is here dramatically softened below the edge of the bulk LA band, due to the strong electron–phonon coupling. Unlike Cu surfaces, surface acoustic modes contribute most to the DW exponent, due to the lack of surface optical modes. The color code in (c) provides the DFT top-layer displacement amplitudes for the surface phonon branches in (b).

The values of the effective mass several times larger than that of Nb are expected for acoustic surface modes, whether RW or SLA, where the displacements decay exponentially and almost in phase inside the lattice, with a decay length proportional to their surface wavelength, but decreasing for increasing spectral localization. As noted above for Cu(110), the softening of the SLA mode below the edge of the bulk LA phonon band is itself an effect of the el-ph interaction. The strong softening in Nb(001), which transforms the SLA branch at wavevectors larger than ∼4 Å^–1^ into a localized flat (Einstein-like) branch below the RW dispersion curve, is consistent with the comparatively large λ_HAS_ that niobium retains at the surface. The fact that by using the Einstein model the effective frequency turns out to correspond to the SLA branch would suggest an equivalent role of the SLA and SV­(RW) branches in the total el-ph interaction at the Nb(001) surface, with contributions from the displacements of more atomic layers beneath the topmost, as indicated, for both branches, by the corresponding effective masses. However, the DFT calculations of the surface el-ph coupling strength by McMillan et al. (Figure 6, middle row of ref 18) definitely favor the Debye model fit.

A similar analysis with eqs 9 and 10 of 2W­(T) for (3 × 1)-O/Nb(001), as reported in Figure 2b of the Méndez et al. paper,^21^ also indicates the Debye model as more appropriate (last line of Table 1). In this case, however, the effective Debye frequency, *ℏ*ω_D_^*^ = 22 meV, corresponds to a good linear fit of the SLA branch in the McMillan et al. DFT calculation, also endowed with a strong el-ph coupling (cfr. Figure 7 of ref 18). It is also remarkable that the effective mass *M*_D_^*^ = 107 a.u. is about the mass of a NbO molecule, which confirms the important role of oxygen in stabilizing the Nb(001) surface, as underlined by McMillan et al., by raising the ultrasoft SLA branch of the clean surface back to the edge of the LA band.

It is finally noted that the value of λ_HAS_ for the clean Nb(001) surface is about one-half of the mass-enhancement factor occurring in the bulk, as discussed by Thompson et al.,^20^ and is indeed consistent with the reduction of the superconducting *T*_c_ observed in thin films.^32^ For the oxidized reconstructed (3 × 1)-O/Nb(001) surface, an even larger reduction to λ_HAS_ = 0.20 ± 0.06 has been derived by Thomson et al.^33^ from the T-dependence of the DW exponent.

In conclusion, it has been shown that in HAS from metal surfaces, where He atoms do not collide directly with surface atoms but through the surface free electron density, the DW exponent receives a major contribution from surface phonons with the largest el-ph coupling. They are in general the surface optical phonons, whose counterphase displacements of atoms within the surface unit cell effects yield the largest surface electron-density oscillations. The Einstein model with a single effective optical frequency and effective mass smaller than the metal atomic mass is shown to provide the appropriate approximation accounting for the temperature dependence of the DW factor for HAS from metal surfaces. This is demonstrated for four different copper surfaces, where the effective Einstein frequency, derived from the experimental T-dependence of the DW exponent, is seen to agree very well with the most relevant surface optical branches.

On the other hand, the classic approximation for the DW factor based on the Debye model, which has been adopted in the literature since the early days of HAS spectroscopy from metal surfaces, is seen to provide for the Cu surfaces too large Debye effective frequencies, much above the known values for bulk copper. It is nevertheless interesting to note that the slope in the long-wave limit of the acoustic mode corresponding to ω_D_^*^ is that of the ubiquitous anomalous longitudinal acoustic resonance, discovered with HAS in practically all metal surfaces as an effect of the surface-enhanced el-ph interaction (Chapter 3 of ref 6). Despite this correspondence, the association of the DW exponent T-dependence for metal surfaces to an Einstein-like optical branch with a large el-ph interaction is far more plausible. Whether this also provides a mechanism for the surface roughening transition observed with He atoms by Lapujoulade et al. in Cu(110) and vicinal surfaces above 500 K^16,17,27^ where λ_HAS_ exhibits a substantial increase,^31^ is worthy of further investigation.

The cases of clean and oxidized (001) surfaces of niobium, provide an instructive scenario, complementary to that of copper surfaces. In niobium—the best elemental superconductor at normal pressure—the el-ph interaction is much larger than that of copper, but at the two above surfaces it appears substantially reduced, due to the lack (or weakness) of surface localized optical phonon branches. For both surfaces the el-ph interaction is shown to be mostly supported by the surface acoustic branches, and therefore better modeled by the classic Debye approximation. Moreover, the Einstein model applied to the clean Nb(001) surface, where the SLA branch is anomalously softened and flat over a large portion of the Brillouin zone, suggests a relevant role of this branch in the el-ph interaction of niobium surfaces.

## Data Availability

Data are contained within the article.

## References

[1] Weinberg W. H. (1972). Application
of Debye-Waller Theory of Atomic and Molecular Scattering from Solid
Surfaces. J. Chem. Phys..

[2] Maradudin, A. A. ; Montroll, E. W. ; Weiss, G. H. Solid State Physics: Theory of Lattice Dynamics in the Harmonic Approximation; Academic Press: 1963; Suppl. 3.

[3] Lovesey, S. W. Theory of Neutron Scattering from Condensed Matter; Oxford University Press: 1987; Vol. 2.

[4] Manson J. R. (1991). Inelastic
Scattering from Surfaces. Phys. Rev. B.

[5] Manson J. R. (1994). Multiphonon
Atom-Surface Scattering. Comput. Phys. Commun..

[6] Benedek, G. ; Toennies, J. P. Atomic-Scale Dynamics at Surfaces - Theory and Experimental Studies with Helium Atom Scattering; Springer-Nature: 2018.

[7] Sklyadneva I. Yu, Benedek G., Chulkov E. V., Echenique P. M., Heid R., Bohnen K.-P., Toennies J. P. (2011). Mode-Selected
Electron-Phonon
Coupling in Superconducting Pb Nanofilms Determined from He Atom Scattering. Phys. Rev. Lett..

[8] Benedek G., Bernasconi M., Bohnen K.-P., Campi D., Chulkov E. V., Echenique P. M., Heid R., Sklyadneva I. Yu, Toennies J. P. (2014). Unveiling mode-selected
electron–phonon interactions
in metal films by helium atom scattering. Phys.
Chem. Chem. Phys..

[9] Manson J. R., Benedek G., Miret-Artés S. (2022). Atom scattering
as a probe of the
surface electron-phonon interaction at conducting surfaces. Surf. Sci. Rep..

[10] Manson J. R., Benedek G., Miret-Artés S. (2016). Electron–Phonon
Coupling Strength at Metal Surfaces Directly Determined from the Helium
Atom Scattering Debye–Waller Factor. J. Phys. Chem. Lett..

[11] Benedek G., Miret-Artés S., Toennies J. P., Manson J. R. (2018). Electron- Phonon
Coupling Constant of Metallic Overlayers from Specular He-Atom Scattering. J. Phys. Chem. Lett..

[12] Benedek G., Miret-Artès S., Manson J. R., Ruckhofer A., Ernst W. E., Tamtögl A. (2020). Origin of the Electron-Phonon Interaction
of Topological Semimetal Surfaces Measured with Helium Atom Scattering. J. Phys. Chem. Lett..

[13] Benedek G., Manson J. R., Miret-Artés S. (2020). The Electron–Phonon Interaction
of Low-Dimensional and Multi-Dimensional Materials from He Atom Scattering. Adv. Mater..

[14] Al
Taleb A., Wan W., Benedek G., Ugeda M. M., Farias D. (2025). Electron-phonon coupling and phonon dynamics in single-layer
NbSe_2_ on graphene: the role of moiré phonons. ACS Nano.

[15] Benedek G., Flach B., Manson J. R., Miret-Artes S., Toennies J. P. (2025). The Electron–Phonon Interaction
at the (110)
Surface of Hydrogen-Covered Mo_1–x_ Re_x_ Alloys from Helium Atom Scattering. J. Phys.
Chem. C.

[16] Lapujoulade J., Lelay Y., Armand G. (1980). The thermal attenuation of coherent
elastic scattering of noble gas from metal surfaces. Surf. Sci..

[17] Lapujoulade J., Perreau J., Kara A. (1983). The thermal
attenuation of elastic
scattering of helium from copper single crystal surfaces. Surf. Sci..

[18] McMillan A. A., Thompson C. J., Kelley M. M., Graham J. D., Arias T. A., Sibener S. J. (2022). A combined helium
atom scattering and density-functional
theory study of the Nb(100) surface oxide reconstruction: Phonon band
structures and vibrational dynamics. J. Chem.
Phys..

[19] Kelley M. M., Sundararaman R., Arias T. A. (2024). A Fully ab Initio Approach to Inelastic
Atom-Surface Scattering. Phys. Rev. Lett..

[20] Thompson C. J., Van Duinen M. F., Kelley M. M., Arias T. A., Sibener S. J. (2024). Correlating
Electron-Phonon Coupling and In-Situ High-Temperature Atomic-Scale
Surface Structure at the Metallic Nb(100) Surface by Helium Atom Scattering
and Density Functional Theory. J. Phys. Chem.
C.

[21] Méndez M., Thompson C. J., Van Duinen M. F., Sibener S. J., Arias T. A. (2025). Ab Initio
Electron-Phonon-Coupling Theory of Elastic Helium-Atom Scattering. Phys. Rev. Lett..

[22] Hulpke E., Hüppauff M., Smilgies D.-M., Kulkarni A. D., de Wette F. W. (1992). Lattice
dynamics of the Nb(001) surface. Phys. Rev.
B.

[23] Bertsch A. V., Chulkov E. V., Eremeev S. V., Lipnitskii A. G., Rusina G. G., Sklyadneva I. Yu. (1995). Vibrations
on the (110) surface of
FCC metals. Vacuum.

[24] Raouafi F., Barreteau C., Desjonquères M.
C., Spanjaard S. (2002). Energetics
of stepped and kinked surfaces of Rh, Pd and Cu from electronic structure
calculations. Surf. Sci..

[25] Chis V., Hellsing B., Benedek G., Bernasconi M., Chulkov E. V., Toennies J. P. (2008). Large Surface Charge-density
Oscillations Induced by Subsurface Phonon Resonances. Phys. Rev. Lett..

[26] Beeby J. L. (1971). The scattering
of helium atoms from surfaces. J. Phys. Part
C Solid State Phys..

[27] Lapujoulade, J. ; Salanon, B. ; Fabre, F. ; Loisel, B. The roughening transition of vicinal surfaces. In Kinetics of Ordering and Growth at surfaces; Lagally, M. G. , Ed.; Plenum Press: 1990.

[28] Chis V., Hellsing B., Benedek G., Bernasconi M., Toennies J. P. (2007). Evidence of longitudinal resonance and optical subsurface
phonons in Al(001). J. Phys.: Condens. Matter.

[29] Benedek G, Bernasconi M, Chis V, Chulkov E, Echenique P M, Hellsing B, Peter Toennies J (2010). Theory of surface
phonons at metal
surfaces: recent Advances. J. Phys.: Condens.
Matter.

[30] Gao K., Cerqueira T. F. T., Sanna A., Fang Y.-W., Dangic D., Errea I., Wan H.-C., Botti S., Marques M. A. L. (2025). The
maximum Tc of conventional superconductors at ambient pressure. Nat. Commun..

[31] Benedek G., Miret-Artés S., Manson J. R., Toennies J. P. (2023). The Electron-Phonon
Interaction at Vicinal Metal Surfaces Measured with Helium-Atom Scattering. Nanomaterials.

[32] Chattaraj A., Anbalagan A. K., Cho J., Liu M. (2026). Materials Studies of
Niobium Thin Films for Quantum Circuit Applications: Progress and
Clallenges. Adv. Quantum Technol..

[33] Thompson C. J., Van Duinen M., Mendez C., Willson S. A., Do V., Arias T. A., Sibener S. J. (2024). Distinguishing the Roles of Atomic-Scale
Surface Structure and Chemical Composition in electron-Phonon Coupling
of the Nb(100) Surface Oxide Reconstruction. J. Chem. Phys. C.

